# Novel insights in health-promoting properties of sweet cherries

**DOI:** 10.1016/j.jff.2020.103945

**Published:** 2020-04-07

**Authors:** Maria Felicia Faienza, Filomena Corbo, Alessia Carocci, Alessia Catalano, Maria Lisa Clodoveo, Maria Grano, David Q.-H. Wang, Gabriele D’Amato, Marilena Muraglia, Carlo Franchini, Giacomina Brunetti, Piero Portincasa

**Affiliations:** a Department of Biomedical Sciences and Human Oncology, Paediatric Section, University of Bari “A. Moro”, Bari, Italy; b Department of Pharmacy-Drug Sciences, University of Bari “Aldo Moro”, Bari, Italy; c Interdisciplinary Department of Medicine, University of Bari “A. Moro”, Bari, Italy; d Department of Emergency and Organ Transplantation, Section of Human Anatomy and Histology, University of Bari “Aldo Moro”, Bari, Italy; e Department of Medicine, Division of Gastroenterology and Liver Diseases, Marion Bessin Liver Research Center, Albert Einstein College of Medicine, Bronx, NY 10461, USA; f Neonatal Intensive Care Unit, Di Venere Hospital Bari, Italy; g Department of Basic and Medical Sciences, Neurosciences and Sense Organs, section of Human Anatomy and Histology, University of Bari “A. Moro”, Bari, Italy; h Clinica Medica “A. Murri”, Department of Biomedical Sciences and Human Oncology, Paediatric Section, University of Bari “A. Moro”, Bari, Italy

**Keywords:** Polyphenol compounds, Antioxidant activity, Cherries, Bioactive compounds, Childhood obesity

## Abstract

Sweet cherry (Prunus avium L.) is one of the most popular and appreciated temperate fruit not only for its sensory and nutritional properties, but also for its content in bioactive compounds. Consumption of sweet cherries brings beneficial effects on to health, which include prevention and modulatory effects in several chronic diseases such as (diabetes mellitus, cancer, cardiovascular and other inflammatory diseases). The presence of natural polyphenolic compounds with high antioxidant potential might drive and partly explain such beneficial effects, but more translational and clinical studies should address this topic. Here, we review the health-promoting properties of cherries and their bioactive compounds against human diseases.

## 1. Introduction

Sweet cherry (*Prunus avium* L.) is one of the most appreciated fruit of the temperate regions, comprising Mediterranean and Central Europe, North Africa, Near and Far East, South Australia and New Zealand, and temperate zones of America (Basanta, de Escalada Plá, Raffo, Stortz, & Rojas, 2014; Mariette et al., 2010). Global sweet cherry production increased over the last 16 years from 1.9 to 2.32 million tons, with Turkey, USA, Iran, as the main producers (Blando & Oomah, 2019). Sweet cherry is an early season fruit mainly consumed as non-processed (Usenik, Fabčič, & Štampar, 2008). The most important indices of cherry quality and maturity that may influence consumer’s acceptance choice are the skin color, sweetness, sourness, firmness, and fruit weight. Skin color relates to fruit ripening and depends on anthocyanin concentration, pH, levels and types of colorless phenolic compounds in the fruits (Serrano, Guillén, Martínez-Romero, Castillo, & Valero, 2005). Other factors include light, temperature, oxygen, metal ions, and enzymes (Delgado-Vargas & Paredes-Lopez, 2002). Sweet cherry contains a moderate amount of carbohydrates, especially simple sugars (e.g., glucose, fructose, sucrose and sorbitol). These components are responsible for sweetness, while sourness is primarily due to the presence of organic acids (e.g., malic, citric, succinic, lactic, and oxalic acids) (Serradilla et al., 2011). Sweet cherry fruits are a source of vitamins, especially vitamin C and minerals, such as potassium, phosphorus, calcium, and magnesium (Schmitz-Eiberger & Blanke, 2012; Yıgıt, Baydas, & Güleryüz, 2009). Also, sweet cherries are enriched with dietary phenolic compounds, including phenolic acids (hydroxycinnamic acids) and flavonoids (anthocyanins, flavan-3-ols and flavonols). Both bring health benefits and play an important role in preventing several chronic diseases related to oxidative stress (Girelli, De Pascali, Del Coco, & Fanizzi, 2016; Picariello, De Vito, Ferranti, Paolucci, & Volpe, 2016). The study of sweet cherries antioxidant activity is therefore gaining growing interest, as a key parameter of fruit quality (Harakotr, Suriharn, Tangwongchai, Scott, & Lertrat, 2014). The fiber content of sweet cherries contributes to the health-promoting characteristics (McCune, Kubota, Stendell-Hollis, & Thomson, 2010). Sweet cherries are also enriched with melatonin, which might function as an antioxidant agent, capable to protect from oxidative stress (Xia et al., 2020; Zhao et al., 2013). A review about the health benefits of sweet cherries (*Prunus avium* L.) has been recently reported (Gonçalves, Bento, Silva, Simões, & Silva, 2019). In this scenario, we aim to discuss novel insights focusing on preclinical and clinical studies about the health promoting properties of sweet cherries. We will also discuss the effects of these cherries on bone impairment associated with childhood obesity.

## 2. Nutrient and bioactive food components of sweet cherries

**Fig. 1.**
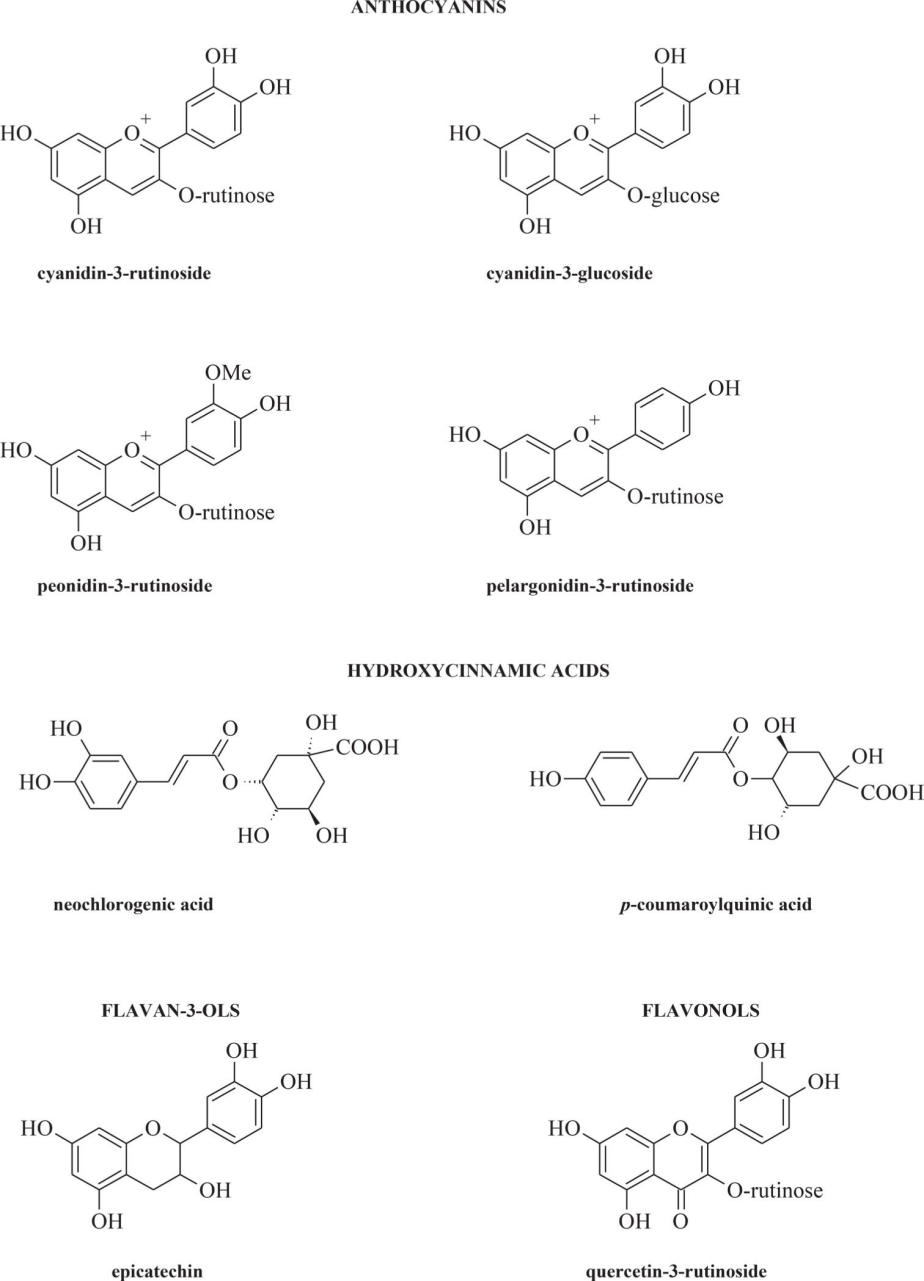
Structures of the major bioactive compounds in sweet cherries.

Sweet cherries contain few calories (63 kcal/100 g), around 80% water, and low content of sodium respect to other minerals such as, for example, potassium. Simple sugar content is low, and ranges from 125 to 265 g/kg of fresh weight and organic acids ranging from 3.67 to 8.66 g/kg of fresh weight (Usenik et al., 2008). Cherries contain both hydrosoluble (C, B) and liposoluble vitamins (A, E and K), some carotenoids (particular *beta*-carotene), and to a lower extent lutein and zeaxanthin (Ferretti, Bacchetti, Belleggia, & Neri, 2010). Minerals include calcium (14 mg/100 g), magnesium (10 mg/100 g), phosphorous (20 mg/100 g) and potassium (200 mg/100 g). Data regarding the nutrient and bioactive food components content of cherries in comparison to other plant foods, as other *Prunus* genus fruits illustrate that sweet cherries are a comparatively good source of fiber, potassium, and in particular anthocyanins. (McCune et al., 2010). Dietary fiber are 2.1 g/100 g, and phenols are present in high amount (approximately 1500 mg total phenols per kg fresh weight). High performance liquid chromatography coupled with photodiode array detector (HPLC-DAD) (Ballistreri et al., 2013) or mass spectrometry detection (Picariello et al., 2016; Bastos et al., 2015; Pacifico et al., 2014; Gonçalves, Ramos, Rosado, Gallardo, & Duarte, 2019) provide the identification and quantification of phenolic compounds. Phenols include hydroxycinnamates, anthocyanins, catechins, and flavonols (Gonçalves, Bento, Silva, & Silva, 2017). Moreover a quantitative metabolomics approach, combining non-targeted mass spectrometry and chemometric analysis, studied six cherry cultivars, and suggested that anthocyanins and colorless phenolic compound contents are cultivar-dependent (Martini, Conte, & Tagliazucchi, 2017). Fig. 1 depicts the most representative bioactive compounds in sweet cherries. Cyanidin-3-rutinoside appears to be the principal anthocyanin in 24 sweet cherry cultivars grown in Sicily (Italy), followed by cyanidin 3-glucoside. Peonidin-3-rutinoside and pelargonidin-3-rutinoside represent minor anthocyanins (Ballistreri et al., 2013). Neochlorogenic acid is the major hydroxycinnamic acid derivative followed by p-coumaroylquinic acid, while chlorogenic acid and ferulic acid appear as small amounts, similarly to hydroxybenzoic acids in sweet cherries. Among flavan-3-ols and flavonols, epicatechin and quercetin-3-rutinoside represent the main compounds belonging to these classes present in sweet cherries (Pacifico et al., 2014). Recently, a total of 40 chlorogenic acids were identified in six cherry cultivars, harvested at commercial maturity in Vignola (Modena province, Italy) during spring or summer, which pointed out hydroxycinnamic acid derivatives as the main class of phenolics by number of compounds (Martini et al., 2017). Hydroxycinnamic acids were also the quantitatively most represented class of phenolic compounds in the cherry cultivars with the exception of two cultivars (Lapins and Durone della Marca) where the most representative class of phenolic compounds were anthocyanins and flavan-3-ols, respectively (Martini et al., 2017).

## 3. Factors affecting sweet cherries composition

Sweet cherry composition changes according to pre-harvest conditions (including cultivar procedures, maturity stage, climate conditions and harvesting timings), and postharvest conditions (including storage and shipping conditions) (Correia, Schouten, Silva, & Gonçalves, 2017). Recently, a review on the potential for supplementary applications of calcium to improve sweet cherry quality at harvest and to extend postharvest shelf life have been reported (Winkler & Knoche, 2019). The stability of phytochemicals and nutritional composition in sweet cherries depends on several factors, including light intensity, temperature, and fruit maturity. Water and nutrient supply to the plant may also influence fruit composition (Ferretti et al., 2010). In general, maturation and ripening are the key physiological factors influencing cherries composition. The rapid increase in size and weight occurs during the last few weeks prior to harvest (Remón, Venturini, Lopez-Buesa, & Burlat, 2003). During this phase of fruit development, formation of major polyphenols occurs (Gonçalves et al., 2007). Serrano et al. (2005) studied antioxidants concentration and activity of sweet cherries at 14 different stages of ripeness. Total anthocyanins increased exponentially from stage 8 and reached their maximum value at stage 14 (63.26 mg cyaniding equivalent activity per 100 g fresh sample). The study suggests that harvesting sweet cherries at stage 12 of ripening, when fruit reaches maximum size would support the development of the highest organoleptic, nutritional, and functional quality attributes. Wang, Jiang, Wang, Jiang, and Feng (2017) found that the early ripening cultivars contained higher free phenolic acids, which was positively related to remarkable antioxidant properties and the inhibition effects on *Alternaria alternata* and tenuazonic acid (TeA) accumulation. However, conjugated phenolics of the late ripening cultivars, mainly including caffeic, 2,3,4-trihydroxybenzoic, *p*-coumaric, and pyrocatechuic acids, achieved the highest antifungal effects and almost completely inhibited the *A. alternata* and TeA production. Sweet cherries are a very perishable fruits with a short shelf life of 7–14 days in conventional cold storage. In many cases, cherries must be sold at low prices to expedite movement and prevent complete losses that can occur once the fruit quality declines below market standards (Padilla-Zakour et al., 2007). Factors such as field conditions, harvest time, rapid cooling, proper refrigeration and packaging, greatly influence the shelf-life and consumer acceptability of the fruit. Proper handling and cooling practices are essential in maintaining sweet cherry quality after harvest, in particular the phenol content of the fruits.

Storage temperature is one the parameter that can affect phenol content in the period of time between the harvesting and the consumption. So many scientific researches study the effect of low temperature storage on the phenol composition of the fruits. Gonçalves et al. (2007) reported the effects of storage temperature and duration on sweet cherry bioactive compounds. The levels of anthocyanins increased during storage being mainly attributable to increases in the cyanidin-3-rutinoside level. Storage at temperatures below zero caused remarkable changes in composition. Changes in total anthocyanin content and antioxidant activity in sweet cherries during frozen storage have been recently reported (Oancea, Draghici, & Ketney, 2016).

Gu et al. in 2020 experimented the cold shock as system to reduce the storage temperature increasing the shelf-life of the cherry fruits. The sweet cherry fruits were immersed in 0 °C ice water (ice:water = 1:1 w/v) for 10 min, then were stored at 0 ± 1 °C and 90% relative humidity (RH) in the dark. The results showed that cold shock treatment not only reduced the weight loss and inhibited the accumulation of malondialdehyde of the sweet cherry fruits, but also maintained some other indicators like firmness, chroma values, and total anthocyanin (Gu et al., 2020).

Considering that post-harvest quality loss on sweet cherry is quite higher, various post-harvest technologies can be applied, combined with low temperature storage, with the aim to preserve or enhance the phenolic content of the fruit. These systems can be physical (ultra-violet light) or chemical (modified atmosphere, hormones or other chemicals).

Postharvest ultra-violet light (UV-C) treatment can reduce postharvest decay in sweet cherries, also if they are stored at room temperature (20 °C) extending the shelf-life of the product. In this study, Pristijono et al. (2017) exposed two sweet cherry cultivars to UV-C light at five different intensities storing the fruits for up to 9 days at 20 °C or 28 days at 1 °C. Results indicated that under certain conditions, postharvest UV-C treatment has the potential to reduce the incidence of decay and maintaining flesh firmness in sweet cherries stored at room temperature (20 °C), however, no data are available on the minor bioactive compounds.

The cherry industry needs to prolong post-harvest shelf life of cherries, to enable long-distance transportation and ensure that fruit keeps quality before going onto the market. Approaches include the optimization of processing, storage, and transport conditions. However, several chemical treatments are potentially harmful to humans and irradiation application is quite limited. The use of modified atmosphere packaging (MAP) is effective in delaying the physico-chemical changes related to quality loss (Habib, Bhat, Dar, & Wani, 2017).

Among the different methods available, modified atmosphere packaging (MAP) is one of the easy applicable in the supply chain. The use of MAP in the large production areas has become more prevalent to extend the life of fresh cherries. MAP, by altering the oxygen and carbon dioxide concentration in the package (3–10% oxygen and 10–15% carbon dioxide), is used to delay the physicochemical changes, to retard microbial spoilage and to retain color by reducing the oxidation, extending the shelf life of sweet cherries, because of it can reduce respiration rates and ripening of fruits. The MAP can also prevent water loss and fruit shriveling by maintaining a high humidity environment of 90–95% relative humidity. The MAP applications that balance the CO_2_ concentrations can also increase the total anthocyanin content of sweet cherry during the cold storage (Padilla-Zakour et al., 2007; Remón, Ferrer, Marquina, Burgos, & Oria, 2000).

The MAP can be combined with other treatment, such as gibberellic acid to improve the reduction of chilling injury. In fact, the use of plant hormones such as gibberellins can be useful for their effect on the slowing of senescence related changes in different fruits.

Öztürk, Ağlar, Karakaya, Saracoğlu, and Gün (2019) demonstrated that pre-harvest gibberellic acid and CaCl_2_ applications are significant to increase the percentage of individual phenolic in fruit, and if associated with MAP, this last factor can reduce the losses of individual phenolic in cold storage.

Giménez et al. (2016) demonstrated the postharvest methyl salicylate (MeSA) treatments can affect quality attributes, bioactive compounds and antioxidant activity of sweet cherries. In fact, MeSA can reduce respiration rate, weight loss, softening, total acidity losses and it can increase in the ripening index during storage at 2 °C for 20 days as compared with non-treated control fruit.

Regard to the total phenolics, total anthocyanins, carotenoids and total antioxidant activity, MeSA was also able to maintain the content of bioactive compounds and antioxidant activity at higher concentrations with respect to control fruit at the end of the storage period.

Recently, the enhancement of quality and antioxidant metabolism of sweet cherry fruit by near-freezing temperature storage (NFTS) has been studied. NFTS was shown to significantly delay and inhibit softening and color change of sweet cherry, to high antioxidant capacity as well as maintain membrane integrity and higher levels of ascorbic acid, sugars and organic acids (Zhao, Liu, Zhang, Cao, & Jiang, 2019).

## 4. Sweet cherries and health-promoting properties

Studies performed *in vitro* and *in vivo* suggest that sweet cherries display anti-inflammatory properties including inhibition of cyclooxygenases COX-1 and COX-2, high antioxidant activity, and low glycemic response. Cherries become sources of bioactive compounds essential to human health and benefits might include anti-carcinogenic properties, prevention of cardiovascular diseases and diabetes. (Kelley, Adkins, & Laugero, 2018). Health promoting effects of sweet-cherries, according to pre-clinical studies, appear in Table 1. The ultimate translational value of such evidences requires further investigations.

### 4.1. Sweet cherries properties

#### 4.1.1. Antioxidant properties

Oxidative stress is one of the main processes underlying human diseases. The overproduction of reactive oxygen species (ROS) leads to cellular damage and inflammation, paving the way to cardiovascular disease, cancer and aging (Poprac et al., 2017). Although the most of studies reporting antioxidant properties of polyphenols have been performed in cell lines or in lab animals, it has been demonstrated, in humans, that the appropriate intake of vegetables and fruits is inversely associated with the risk of many chronic diseases associated to increased of ROS (Zhang et al., 2015).

Anthocyanins are responsible for the red–purple color in fresh sweet cherries, and have a potent antioxidant activity *in vitro*. This effect reduces ROS production and cellular oxidative stress damage.

A recent *in vitro* study confirmed that a phenolic-rich extract (cyanidin-3-rutinoside, cyanidin-3-glucoside, peonidin-3-glucoside and neochlorogenic acid) obtained by a Portuguese variety of cherry (Saco Cherry), had a potent antioxidant role (Matias et al., 2016). Compounds had free radical scavenging activity in intestinal epithelial and neuronal cells, and were an interesting source for the prevention of oxidative stress-induced disorders such as intestinal inflammation disorders or neurodegenerative diseases.

Some sweet cherry cultivars (*Prunus avium* L.) grown on the mountainsides of the Etna volcano (Sicily, Italy), also contain amounts of phenolic compounds with antioxidant capacity (Ballistreri et al., 2013).

Among the phenolic compounds, the main anthocyanins in the Italian sweet cherry cultivar Ferrovia were cyanidin-3-rutinoside and cyanidin-3-glucoside (Crupi, Genghi, & Antonacci, 2014).

Cyanidin and cyanidin-3-glucoside have a protective effect on DNA cleavage, a dose-dependent free radical scavenging activity, and a significant inhibition of xanthine oxidase activity (Acquaviva et al., 2003).

The potent antioxidant activity of sweet-cherry has been studied also *in vivo*.

Rats exposed to hepatic ischemia–reperfusion (I/R) mimic an oxidative stress model. A 14-day diet enriched in cyanidin 3-glucoside significantly suppressed liver damage caused by hepatic I/R (Tsuda, Horio, & Osawa, 2000).

In another study, rats fed with vitamin E–deficient diets for 12 weeks received purified anthocyanin-rich extracts. The anthocyanin diet improved plasma antioxidant capacity and reduced the level of hydroperoxides and 8-oxo-deoxyguanosine (markers of lipid peroxidation and DNA damage following vitamin E deficiency) (Ramirez-Tortosa et al., 2001).

Cultivars of *Prunus avium* containing high levels of anthocyanins showed greater bioprotective capacity compared to other cultivars. The protection of human cells from oxidative stress was stronger than the protection by vitamin C (Leong, Burritt, Hocquel, Penberthy, & Oey, 2017).

#### 4.1.2. Anticarcinogenic activity

Cherries contain phytocompounds (in particular phenols) which are higher than amounts found in several other fruits, although different cultivars display variability. Factors affecting the content and stability of phytochemicals include the pre-harvest temperature, light intensity, fruits maturity, and type of consumption. Sweet cherries, as an example, are mainly consumed as fresh fruit with increased beneficial properties.

Anthocyanins, particularly cyanidin-3-glucoside, display anticancer activity through multiple pathways (Duthie, 2007). Mechanisms include antimutagenic activity (Ohara, Matsuhisa, Hosokawa, & Mori, 2004; Yoshimoto, Okuno, Yamaguchi, & Yamakawa, 2001), cell cycle arrest (Renis et al., 2008), induction of apoptosis (Shih, Yeh, & Yen, 2005; Yi, Fischer, Krewer, & Akoh, 2005), angiogenesis (Bagchi, Zafra-Stone, Losso, Sen, Roy, Hazra, & Bagchi, 2007), inhibition of oxidative DNA damage (Singletary, Jung, & Giusti, 2007), inhibition of COX-2 enzymes, inhibition of carcinogen activation, and induction of phase II enzymes for detoxification (Shih, Yeh, & Yen, 2007; Srivastava, Akoh, Fischer, & Krewer, 2007). Cyanidin-3-glucoside has also a potent inhibitory effect on cell growth via G2/M arrest which has been associated with reduction of the CDK-1, CDK-2, cyclin B1 and cyclin D1 levels and increase of caspase-3 activation, chromatin condensation and cell death (Chen et al., 2005). Cell lines exposed to sweet cherry anthocyanins displayed inhibition of proliferation and induction of apoptosis (Chen et al., 2005). Cyanidin may reduce the risk for malignant transformation by promoting cellular differentiation (Serafino et al., 2004). Recently, the anticancer properties of sweet cherry extract on human prostate cells have been studied. The sweet cherry extract diminished the viability of neoplastic and non-neoplastic cell lines (Silva et al., 2019).

#### 4.1.3. Anti-inflammatory activity

Inflammation is a complex biological process in response to tissue injury. Inflammatory cells provide a microenvironment advantageous for tumor development, and therefore anti-inflammatory therapy can prevent early neoplastic progression and malignant conversion (Coussens & Werb, 2002). Seeram et al. (Seeram, Momin, Nair, & Bourquin, 2001) investigated the anti-inflammatory effects of cyanidin alone, and anthocyanins from a wide variety of cherries. Sweet cherries inhibited COX-1 and COX-2 enzyme activity by an average of 28% and 47%, respectively. The cyclooxygenase inhibitory activities of anthocyanins from raspberries and sweet cherries were comparable to those of ibuprofen and naproxen at 10 μM concentrations.

Anthocyanins from sweet cherries showed a significant COX-2 inhibitory effect related to down-stream inhibition of mitogen-activated protein kinase (MAPK) (Hou, Yanagita, Uto, Masuzaki, & Fujii, 2005).

Cherries reduced the inflammatory response in rats with inflammation-related chronic illness (He et al., 2006).

A pilot study investigated the effects of consuming sweet cherries on plasma lipids and markers of inflammation in healthy humans. Sweet cherries had a selective modulatory effect on some markers of inflammation, such as protein C reactive (Kelley & Kelley, 2006). A study checked for the anti-inflammatory properties of sweet cherry components in relation to pain control (Tall & Raja, 2004).

### 4.2. Sweet cherries health-promoting effects

#### 4.2.1. Prevention of cardiovascular diseases

The exposition of endothelial cells isolated from bovine arteries to cyanidin-3-glycoside for several hours increased nitric oxide output, reduced local oxidative stress, and vascular inflammation. The formation of foam cells (precursors for the development of atherosclerotic plaque) also decreased (Xu, Ikeda, & Yamori, 2004).

A study on mice foam cells exposed to doses of cyanidin-3-glycoside showed that cholesterol was removed from macrophages in a dose-dependent manner. This finding suggests a protective effect of cyanidin in reducing cardiovascular risk (Xia et al., 2005)

#### 4.2.2. Control of diabetes

Anthocyanins may reduce insulin resistance and glucose intolerance (Al-Awwadi et al., 2005). The antioxidant activity of anthocyanins may protect pancreatic β-cells from glucose-induced oxidative stress and associated complications of diabetes (Gonçalves et al., 2017). In a study on cell culture, anthocyanins and anthocyanidins from sweet cherries were combined with several glucose loads. The anthocyanin and anthocyanidin-enriched cells exibited a significant enhancement in insulin secretion, compared to control (Ghosh & Konishi, 2007; Jayaprakasam, Vareed, Olson, & Nair, 2005).

The role of anthocyanins in the glycemic control was also studied in two mouse models (Jayaprakasam, Olson, Schutzki, Tai, & Nair, 2006). High fat diets induced obesity and hyperglycemia. The supplemental feedings of cherries had protective effects, namely decreased triglyceride synthesis, glucose and leptin levels. Notably, the glycemic index of sweet cherries is generally lower than the glycemic index of other fruits such as apricot, grapes, peach and blueberry (Foster-Powell, Holt, & Brand-Miller, 2002). A lower glycemic index in response to sweet cherry consumption can depend on the glucose-lowering effects of fiber content of cherries. The lower glycemic index makes sweet cherries a better fruit-based snack food in diabetic patients.

#### 4.2.3. Effects on gout

Consumption of sweet cherry reduces serum levels of urate in healthy women (Jacob et al., 2003), and suggests a potential role of cherries for the treatment of gout (Petraccia, Fraioli, Liberati, Lopalco, & Grassi, 2008). Indeed, it might modify the course of established gout (Gelber & Solomon, 2012; Zhang et al., 2012). In established gout, cherry ingestion could decrease the recurrence of gout flares (Zhang et al., 2012). In particular, authors examined 633 patients with gout and at least one gout flare in the preceding year. Patients completed study questionnaires and one-year follow-up to record cherry use, triggers for flare, and characteristics of the flare. Over 40% of the patients used fresh cherries in their diets (more than cherry extract). Looking at the total number of flares within the observation period, cherry intake over a two-day period prior the gout attack yielded 35% lower risk of gout attacks compared with no intake of cherry. Moreover, the flare risk tended to decrease with increased cherry consumption (up to three servings, meaning 10–12 cherries per serving over two days). The beneficial effect of cherry intake on gout flare was independent of gender and body size. Reducing some risk factors (i.e. purine or alcohol consumption) also decreased the beneficial effects of cherries, while allopurinol or colchicine use increased the beneficial effects of cherry consumption. Cherry intake is therefore associated with a lower risk of gout attacks. Further studies need to investigate which specific component, and to which extent the effect is long-term reproducible.

#### 4.2.4. Effects on control of body weight and bone impairment associated with childhood obesity

Obesity is one of the most important global health problems (Haslam & James, 2005). Even in childhood, obesity contributes to the development of metabolic and cardiovascular diseases (Faienza et al., 2019, 2012, 2013; Faienza, Wang, Fruhbeck, Garruti, & Portincasa, 2016; Gilbert & Slingerland, 2013; Nacci et al., 2013). Current anti-obesity pharmacological treatments have limitations, i.e. adverse effects and high rates of secondary failure (Kang & Park, 2012). I*n vitro* and experimental models point to the effects of polyphenols on obesity and related metabolic disorders. Among the others, polyphenols induce satiety, stimulate energy expenditure, inhibit adipocyte differentiation, promote adipocyte apoptosis, modulate lipolysis, and activate oxidation (Costa, Garcia-Diaz, Jimenez, & Silva, 2013; Meydani & Hasan, 2010; Wu et al., 2014; Yun, 2010). In detail, sweet cherry anthocyanins decrease adipocyte size, leptin secretion, serum triglyceride, glucose, total cholesterol, liver triglycerides, and LDL-cholesterol. These effects are associated to decreased expression of IL-6 and TNFα genes.

Childhood obesity is also associated with high incidence of bone fractures (Rana et al., 2009). Animal models point to a relationship between childhood obesity and bone impairment. Mice fed with HFD-diet have bone loss due to high osteoclastic bone resorption, mediated by the increase of pro-osteoclastogenic cytokines and pre-osteoclasts in the bone marrow microenvironment (Shu et al., 2015). Antioxidant compounds might act as anti-resorption therapies while reducing the osteoclast activity without inducing their apoptosis. This step restores physiological bone remodeling (Domazetovic, Marcucci, Iantomasi, Brandi, & Vincenzini, 2017), Furthermore, tea and dried plum polyphenols *in vitro* inhibit osteoclastogenesis (Bu et al., 2008).

**Fig. 2.**
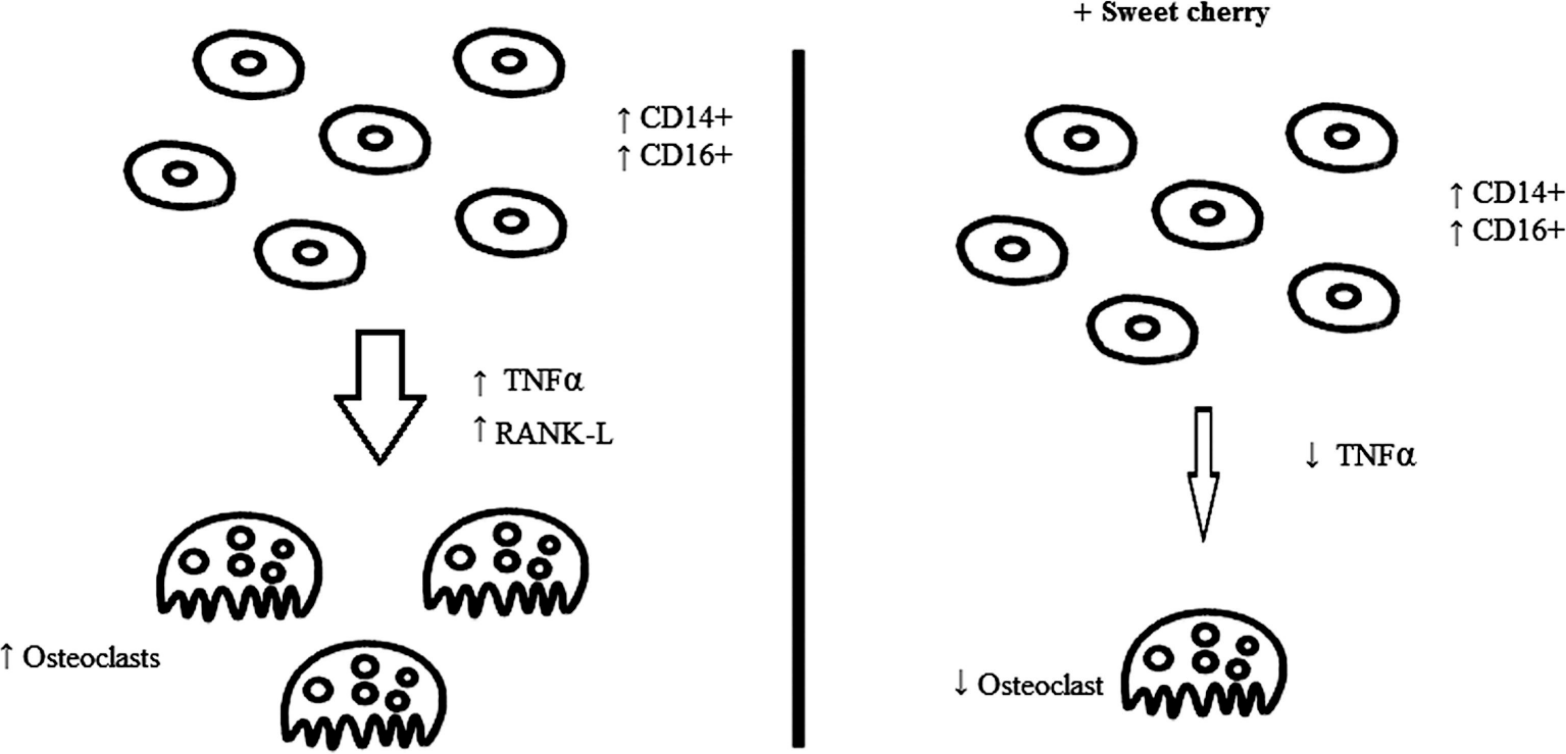
Osteoclastogenesis in cultures of peripheral blood mononuclear cells (PBMCs) from obese children mediated by TNF-α and RANKL, and effects of sweet cherries.

Recently, we reported that sweet cherry extracts, dose-dependently reduced spontaneous formation of multinucleated osteoclasts in cultured peripheral blood mononuclear cells (PBMCs) from obese children. The experiment did not affect cell viability. The spontaneous osteoclastogenesis occurred with high percentage of circulating CD14+/CD16+ cells and high levels of RANKL and TNFα (Fig. 2). A 24 h treatment of obese PBMCs with sweet cherry extracts determined a significant reduction of TNFα expression. These evidenced pave the way to the use of cherry extracts as nutraceutical food in obesity (Corbo et al., 2019).

#### 4.2.5. Liver steatosis

Liver steatosis, especially the nonalcoholic fatty liver disease (NAFLD) and its progressive form, nonalcoholic steatohepatitis (NASH), the hepatic expression of the metabolic syndrome (Shu et al., 2015), represents an emerging problem worldwide. Supplementation with sweet cherry anthocyanins protected mice from high-fat diet-induced hepatic steatosis. The effect involved hepatic gene expression profiles (> 1000) contributing to 16 pathways (PPAR signaling pathway, fatty acid metabolism, steroid biosynthesis, and biosynthesis of unsaturated fatty acids) (Domazetovic et al., 2017). A similar effect occurred with tart cherries as a model of anthocyanin-rich foods, in the Dahl Salt-Sensitive rat (developing insulin resistance and hyperlipidemia). The supplemented diet with whole tart cherry during 90 days caused enhanced hepatic PPAR-alpha mRNA, enhanced hepatic PPAR-alpha target acyl-coenzyme A oxidase mRNA activity, and increased plasma antioxidant capacity. This molecular action was associated with reduced hyperlipidemia, fasting blood glucose, hyperinsulinemia, and reduced fatty liver (Bu et al., 2008). Of note, sweet cherry acting as a functional fruit promote active glucose consumption by HepG2 cells (Cao et al., 2015). The effect was distinctively mediated by three fractions, anthocyanin rich fraction, hydrocinnamic acid rich fraction, and flavonol rich fraction (Corbo et al., 2019). As sweet cherries are a rich source of dietary phenolic compounds with antioxidant capacity, a general beneficial effect is anticipated in NAFLD, but further clinical studies are necessary to clarify this important issue (Wang, Portincasa, & Neuschwander-Tetri, 2011).

#### 4.2.6. Additional potential health benefits

Melatonin is a natural hormone secreted by the pineal gland, which modulates a wide variety of physiological functions. Besides the well-known chronobiotic and sleep inducing properties, melatonin might have many other effects, including an antitumor, anti-inflammatory, neuroprotective, and antioxidant effect, and also acting as pain modulator, (Song et al., 2016). Sweet cherry are rich in melatonin. (González-Gómez et al., 2009) studied the melatonin content in eight different sweet cherry cultivars by high performance liquid chromatography with mass spectrometry detection (HPLC-MS). Melatonin levels in ripen cherries were higher than in unripe and intermediate ripe ones, in some of which it was even undetectable Melatonin in cherry fruits could act as an antioxidant to protect from oxidative stress. Importantly, plant tryptophan decaboxylase gene (PaTDC), whose expression is related to melatonin production in cherries, was identified in cherry fruits (Zhao et al., 2013). Sweet cherries also have antimicrobial activity in the defense against pathogens as gram-positive and gram-negative bacteria and *Candida albicans* (Seymour et al., 2008; Wang et al., 2017). In particular, sweet cherry stems have a high content of sakuranetin, ferulic acid, p-coumaric acid, p-coumaroylquinic acid, chlorogenic acid and its isomer neochlorogenic acid. Thus, cherry stems can be further exploited to purify compounds and produce coproducts for pharmaceutical industry.

## 5. Conclusions

Sweet cherries are pleasant fruits in the human diet, and accumulate important nutrients and bioactive food components, as well. They contain high amounts of phytocompounds, in particular of phenols when compared to several other fruits, but with high variability among different cultivars. Besides providing essential vitamins, minerals, carotenoids and dietary fiber, cherries contain of bioactive food components supports their potential preventive health benefits, thus, their supplementation in our daily diet should be recommended as it can potentially reduce the risk of health problems. A number of studies exist regarding health-promoting effects of cherries, especially in cellular and animal models. Sweet cherries also possess antioxidant, anti-inflammatory, anti-carcinogenic properties, besides prevention activity for cardiovascular disease and diabetes. Thus, given the huge potentiality of sweet cherry in mitigating health issues, the whole consumption of this fruit should be prescribed. This will provide the consumers other important ingredients like fibers, vitamins and minerals, etc. Moreover, sweet cherry is preferable to other fruits (apricot, grapes, peach and blueberry) due to its lower glycemic index. The field is therefore intriguing and challenging, but further studies at a more translational level urge to dissect the complex mechanisms linking consumption of sweet cherries (by different types and amounts) to health promoting effects.

## Figures and Tables

**Table 1:** In *vitro* and *in vivo* studies investigating health promoting effects of sweet cherries.

Type of study	Health promoting effects	Source	References
**In vitro** – intestinal epithelial cells (Caco-2 cells) – neuronal cells (SK-N-MC cells) – human gastric adenocarcinoma cells – pancreatic beta-cells (INS-1 832/13) – lipopolysaccharide (LPS)-activated murine macrophage RAW264 cells – HepG2 Cells	– anti-oxidant properties – inhibition of proliferation and induction of apoptosis – enhancement in insulin secretion – anti-Cox 2 effects – activation of glucose consumption	Saco cherry Prunus avium Anthocyanins	Matias et al., 2016; Leong et al., 2017; Shih et al., 2005; Jayaprakasam et al., 2005; Hou et al., 2005; Cao et al., 2015; Corbo et al., 2019
***In vivo*** Rats Mice C57BL/6 Mice Freund’s adjuvant-induced arthritis in rats	– anti-oxidant properties – reduction of atherosclerotic plaque – amelioration of obesity and glucose intolerance – anti-inflammatory -anti-obesity effects – reduction of liver steatosis	Anthocyanins Anthocyanidins Cornelian cherry (Cornus mas)	Tsuda et al., 2000; Ramirez-Tortosa et al., 2001; He et al., 2006; Xia et al., 2005; Jayaprakasam et al., 2006; Song et al., 2016
***Humans*** Healthy women Obese children	– anti-inflammatory and anti-pain effects – reduction of plasma urate levels – effects on bone health	Sweet cherries (Bigarreaux, Ferrovia, Georgia)	Kelley et al., 2006; Jacob et al., 2003; Petraccia et al., 2008; Gelber et al., 2012; Zhang et al., 2012
